# Cytotoxic (salen)ruthenium(iii) anticancer complexes exhibit different modes of cell death directed by axial ligands†
†Electronic supplementary information (ESI) available. CCDC 1530521 and 1530522. For ESI and crystallographic data in CIF or other electronic format see DOI: 10.1039/c7sc02205k



**DOI:** 10.1039/c7sc02205k

**Published:** 2017-07-31

**Authors:** Cai Li, Kwok-Wa Ip, Wai-Lun Man, Dan Song, Ming-Liang He, Shek-Man Yiu, Tai-Chu Lau, Guangyu Zhu

**Affiliations:** a Department of Chemistry , City University of Hong Kong , 83 Tat Chee Ave , Kowloon Tong , Hong Kong SAR . Email: bhtclau@cityu.edu.hk ; Email: guangzhu@cityu.edu.hk; b Department of Biomedical Sciences , City University of Hong Kong , 83 Tat Chee Ave , Kowloon Tong , Hong Kong SAR; c City University of Hong Kong Shenzhen Research Institute , Shenzhen , P. R. China; d Institute of Molecular Functional Materials , City University of Hong Kong , 83 Tat Chee Ave , Kowloon Tong , Hong Kong SAR

## Abstract

Two novel series of (salen)ruthenium(iii) complexes bearing guanidine and amidine axial ligands were synthesized, characterized, and evaluated for anticancer activity. *In vitro* cytotoxicity tests demonstrate that these complexes are cytotoxic against various cancer cell lines and the leading complexes have remarkable cancer-cell selectivity. A detailed study of the guanidine complex **7** and the amidine complex **13** reveals two distinguished modes of action. Complex **7** weakly binds to DNA and induces DNA damage, cell cycle arrest, and typical apoptosis pathways in MCF-7 cells. In contrast, complex **13** induces paraptosis-like cell death hallmarked by massive vacuole formation, mitochondrial swelling, and ER stress, resulting in significant cytotoxicity against human breast cancer cells. Our results provide an extraordinary example of tuning the mechanism of action of (salen)ruthenium(iii) anticancer complexes by modifying the structure of the axial ligands.

## Abstract 2

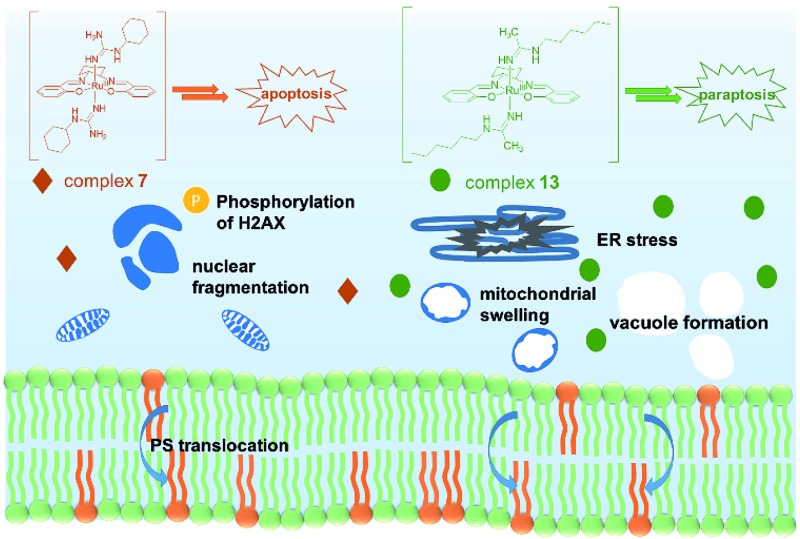
A cancer-cell selective bis(guanidine)-ruthenium(iii) complex induces apoptosis, whereas its amidine analogue effectively kills cancer cells through paraptosis pathways.

## Introduction

Platinum drugs are among the most commonly used chemotherapeutic regimens; however, issues including drug resistance, undesirable side effects, and high toxicity have led to a call for alternatives.^1,2^ Many ruthenium compounds have shown promising anti-tumor properties, especially the leading complexes NAMI-A {(ImH)[*trans*-Ru(DMSO)(Im)Cl_4_], Im = imidazole} and KP1019 {(IndH)[*trans*-Ru(Ind)_2_Cl_4_], Ind = indazole} that are currently undergoing clinical trials; a number of (arene)ruthenium(ii) complexes are also in the pipeline.^3–10^

Guanidium-rich compounds have received considerable attention recently because of their incredible cell-penetrating ability. They act as molecular transporters for carrying drugs and probes across biochemical barriers.^11^ Moreover, the highly versatile guanidine ligands can provide a platform for the facile tuning of the solubility, redox potential, hydrogen bonding, and lipophilicity of metal complexes, which would ultimately enhance their biological activity.^12–15^ A number of metal complexes bearing guanidine ligands have been reported, and the guanidine groups endow some compounds with therapeutic properties.^16^ For example, a series of guanidine-platinum(ii) complexes were reported to target DNA and display anticancer activity.^15^ Copper(ii) complexes containing guanidine were found to interact with proteins and induce cancer cell death.^17^ Incorporation of a biologically compatible guanidine ligand onto a ruthenium platform may offer effective anticancer activity; however, so far there are no examples of ruthenium guanidine complexes.

Herein, we report the synthesis, characterization, cytotoxicity, and mechanistic investigation of two novel series of complexes bearing guanidine and amidine axial ligands. Our studies provide not only a new class of (salen)ruthenium(iii) anticancer complexes but also an example of controlling cell death pathways by tuning the structure of the axial ligands in these (salen)ruthenium(iii) complexes.

## Results and discussion

**Scheme 1:**
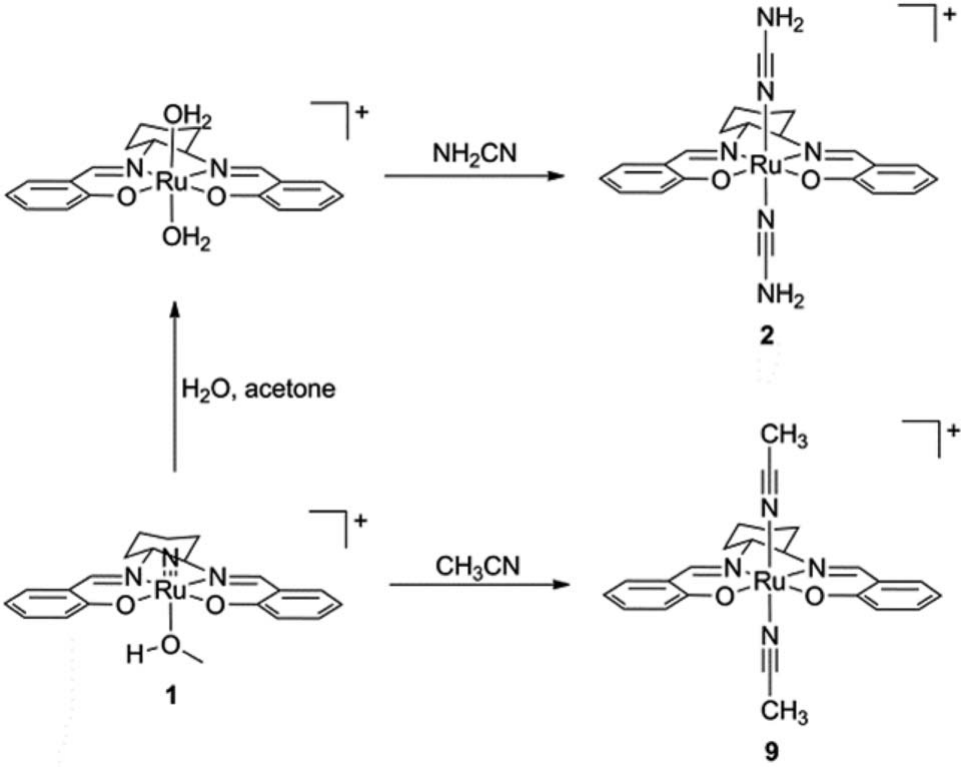
Preparation of (salen)ruthenium(iii) cyanamide and nitrile complexes.

**Fig. 1:**
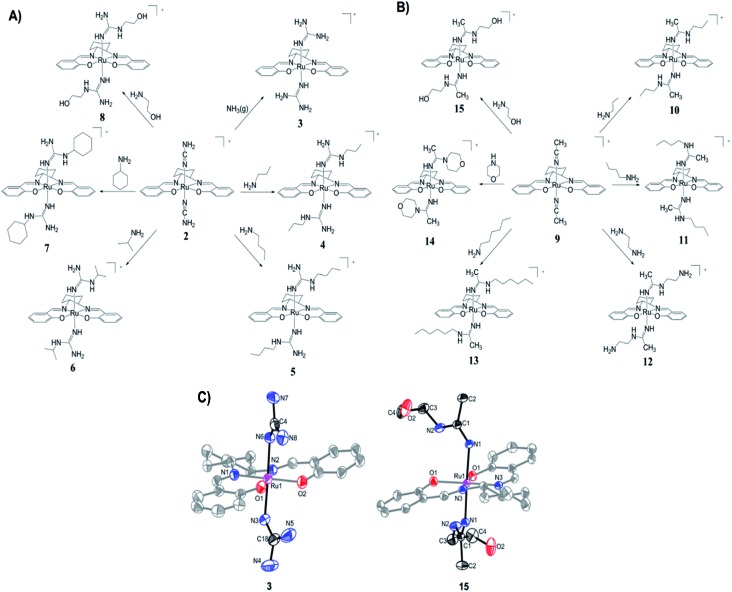
Synthesis of (A) Ru(iii) guanidine complexes **3–8** and (B) Ru(iii) amidine complexes **10–15**. (C) ORTEP structures of the cations of complexes **3** and **15**. Hydrogen atoms have been omitted for clarity.

The preparation of (salen)ruthenium(iii) cyanamide (**2**) and nitrile (**9**) precursors is summarized in Scheme 1, using the (salen)ruthenium(vi) nitrido complex (**1**) as the starting material.^18,19^ Two series of bis(guanidine)- and bis(amidine)ruthenium(iii) complexes were then synthesized *via* nucleophilic addition of various amines to complexes **2** (Fig. 1A) and **9** (Fig. 1B), respectively.^20^

Complexes **3–8** and **10–15** are paramagnetic with room-temperature magnetic moments of 1.88–2.09 *μ*_B_ (solid sample, Gouy method), which is consistent with d^5^ Ru^III^ complexes with one unpaired electron. The cyclic voltammograms of these complexes show two reversible couples, in the range of +0.76 to +0.23 V and –0.87 to –1.32 V *versus* ferrocene, which are assigned to metal-centered Ru^IV/III^ and Ru^III/II^ couples, respectively (Fig. S1†). The oxidation potentials are assigned as metal-based because they are sensitive to the nature of the axial ligand (ranging from +0.23 to +0.76 V). Similar assignments for other (salen)ruthenium(iii) complexes have also been reported in the literature.^18^ The redox potentials and IR (KBr) data are summarized in Table S1.†

The X-ray crystal structures of complexes **3** and **15** have been determined (Fig. 1C and Tables S2–S4†). Both complexes have a distorted octahedral geometry with the tetradentate salen ligand in the equatorial plane. The axial positions are occupied by two guanidine ligands in **3** and two amidine ligands in **15**.

The cytotoxicity profiles of the ruthenium complexes were assessed using several human carcinoma cell lines including HeLa (cervical), A549 (lung), MCF-7 (breast), and HepG2 (liver), which are typically used for the biological evaluation of metal-based drugs.^21,22^ In general, most of the ruthenium complexes are cytotoxic against different types of cancer cells, with IC_50_ values ranging from submicromolar to micromolar levels (Table 1). Among the guanidine complexes, **7** is the most cytotoxic one with an IC_50_ value of 3.3 μM in MCF-7 cells. Among the amidine complexes, complex **13** is the most active one and displays significantly improved cytotoxic potency compared to cisplatin. The IC_50_ values of complex **13** are as low as 0.1 and 0.5 μM in HeLa and MCF-7 cells, respectively, showing 56- and 24-fold increases over those of cisplatin, respectively. We further tested some of the leading complexes in the human ovarian A2780 cell line and cisplatin-resistant A2780cisR cell line. Notably, the tested ruthenium complexes display similar cytotoxic potency towards sensitive and resistant cells, showing negative cross-resistance with cisplatin. We also measured their cytotoxicity in MRC-5 human normal lung fibroblasts. Remarkably, the guanidine complex **7** is inactive in the normal cells with IC_50_ values of >100 μM, indicating its impressive cancer-cell selectivity. In addition, the amidine complexes **11** and **13** show marginal selectivity towards lung carcinoma cells over the normal cells. For example, the IC_50_ values of complex **13** in A549 and MRC-5 cells are 0.3 and 1.8 μM, respectively. In contrast, cisplatin shows identical low-micromolar cytotoxicities against both cancer and normal cells. The selectivity index increases from 0.9 for cisplatin to 6.0 for complex **13**. The improved cancer-cell selectivity of the leading complexes distinguishes them from other metal compounds and makes them valuable for further evaluation and development. The leading guanidine complex **7** and amidine complex **13** are proven to be stable in both aqueous and culture media, since no peak shifting from the UV-vis spectra is observed after incubation for 8 h (Fig. S2†).

**Table 1:** IC_50_ values (μM) of guanidine-ruthenium(iii) complexes **3–8**, amidine-ruthenium(iii) complexes **10–15**, and cisplatin. Cells were treated for 72 h and cell viability was determined by the MTT assay

Complex	HeLa	A549	MCF-7	HepG2	A2780	A2780cisR	RF^*a*^	MRC-5	SI^*b*^
**3**	38.1 ± 2.1	47.8 ± 14.6	16.1 ± 1.1	37.0 ± 0.4	—	—		—	—
**4**	27.1 ± 0.7	43.9 ± 3.8	15.0 ± 0.7	29.4 ± 2.1	—	—		—	—
**5**	7.3 ± 0.6	14.3 ± 0.6	4.3 ± 0.2	15.7 ± 0.2	11.2 ± 1.8	13.5 ± 1.6	1.2	1.1 ± 0.2	0.1
**6**	54.9 ± 4.2	72.8 ± 10.1	36.2 ± 3.3	53.3 ± 6.1	—	—		—	—
**7**	3.4 ± 0.3	6.6 ± 0.5	3.3 ± 0.3	7.7 ± 1.1	4.9 ± 0.2	4.0 ± 0.5	0.8	>100	>15.2
**8**	>100	>100	>100	>100	—	—		—	—
**10**	10 ± 0.8	14.2 ± 0.7	16.0 ± 0.6	5.3 ± 0.9	—	—		—	—
**11**	2.4 ± 0.1	2.7 ± 0.7	1.8 ± 0.2	3.4 ± 0.1	4.1 ± 0.4	4.0 ± 1.4	1.0	9.2 ± 2.6	3.4
**12**	>100	>100	>100	>100	—	—		—	—
**13**	0.1 ± 0.003	0.3 ± 0.03	0.5 ± 0.04	0.6 ± 0.05	0.3 ± 0.01	0.2 ± 0.05	0.7	1.8 ± 0.2	6.0
**14**	57.9 ± 3.3	45.9 ± 3.7	44.4 ± 8.0	21.6 ± 2.5	—	—		—	—
**15**	>100	>100	>100	>100	—	—		—	—
Cisplatin	5.6 ± 0.1	2.9 ± 0.3	12.2 ± 1.6	2.6 ± 0.1	1.8 ± 0.6	11.6 ± 0.6	6.4	2.6 ± 0.3	0.9

^*a*^RF (resistant factor) is defined as IC_50_ in A2780cisR/IC_50_ in A2780.

^*b*^SI (selectivity index) is defined as IC_50_ in MRC-5/IC_50_ in A549.

To reveal the possible reasons for the significant cytotoxic potency of complexes **7** and **13**, the most active ones in the guanidine and amidine series, respectively, their mechanisms of action were subsequently scrutinized. To test whether DNA is their potential target, the complexes were first tested for their binding affinity for calf thymus DNA (CT-DNA). Our data indicate that 76% of cisplatin in the reaction binds to CT-DNA after incubation overnight, which is consistent with other reports (Fig. S3A†).^23,24^ Complex **7** weakly binds to CT-DNA, although not as efficiently as cisplatin. In contrast, complex **13** shows no affinity to CT-DNA. In addition, complex **13** does not affect the mobility of plasmid DNA (Fig. S3B†). Therefore, complex **13** may not induce its cytotoxic action through DNA-damaging pathways.

Additional cell-based assays were carried out to evaluate the complexes. MCF-7 cells were used since this cell line is poorly responsive to cisplatin but is the most responsive one to the guanidine complexes. Firstly, cellular accumulation and distribution of the complexes were evaluated. MCF-7 cells were treated with 10 μM complex **7** or **13** for 8 h, followed by the measurement of ruthenium levels in the whole cells as well as in the membrane, cytoplasm, and nuclei. The accumulation levels of ruthenium for complexes **7** and **13** in the whole cells were 57.5 and 547 ng Ru per 10^6^ cells, respectively (Fig. S4†). Therefore, complex **13** has a 9.5-fold increase in cellular accumulation compared to complex **7**, and this elevated uptake may contribute to its higher cytotoxicity. The cellular distribution of the ruthenium complexes was analyzed as well. After the cellular entrance, the majority of the ruthenium compounds are localized in the cytoplasm, and the cytoplasmic fractions of complexes **7** and **13** are 72% and 86%, respectively. Only 3% and 2% of the complexes are found in the nuclear region for complexes **7** and **13**, respectively (Fig. S4†). These data indicate that the ruthenium complexes can efficiently enter cells, accumulate in the cytoplasm but not in the nuclear region, and execute their cytotoxic effects.

The ability of the complexes to arrest the cell cycle was studied. Upon 24 h of treatment, cisplatin displays strong S phase block as previously reported.^25^ Complex **7** weakly arrests the cell cycle at the G_2_/M phase. For instance, upon treatment with 5 μM complex **7**, the fraction of cells in the G_2_/M phase slightly increases from 11.2% to 17.6% (Fig. S5†). In contrast, complex **13** does not induce detectable cell cycle arrest even at its IC_70_ value, indicating it may not act as a DNA-damaging agent.^26,27^ Thus, although the ruthenium complex **13** is able to efficiently enter cancer cells and effectively kill them, it is not able to arrest the cell cycle, at least at the concentrations tested, evidently confirming that complex **13** induces cell death in a different way to complex **7**.

**Fig. 2:**
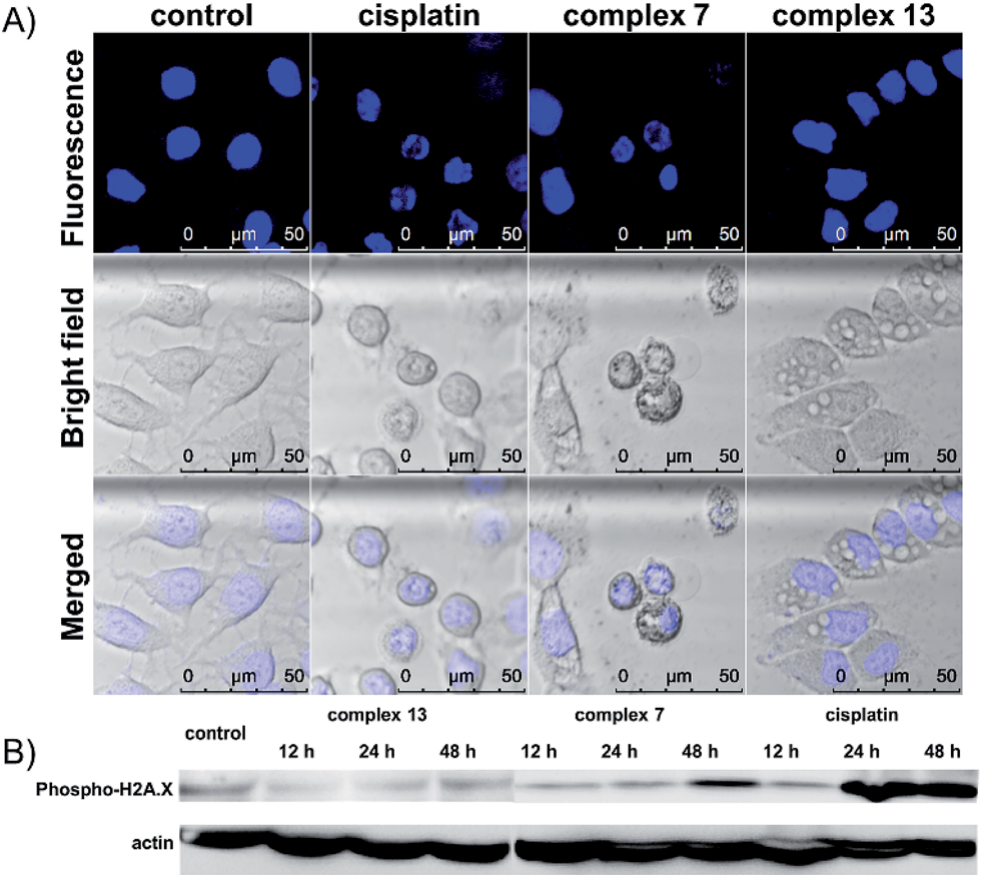
(A) Confocal images of Hoechst stained MCF-7 cells upon treatment with cisplatin, complex **7**, or complex **13** at their IC_70_ values for 24 h. (B) Western blot analysis of DNA damage marker γ-H2AX in MCF-7 cells upon treatment with complex **13**, complex **7**, or cisplatin at their IC_70_ values for different periods of time.

The cell death pathway upon treatment with the (salen)ruthenium(iii) complexes was further investigated using an Annexin V/7-AAD double staining assay. MCF-7 cells were exposed to cisplatin or the ruthenium compounds at their IC_70_ or IC_50_ values for 24 h (Fig. S6†). At the IC_70_ values, 6.9% and 13.1% of dead cells (Annexin V+/7-AAD+) are induced by complex **7** and cisplatin, respectively. Notably, 24.2% of MCF-7 cells treated with complex **13** were dead cells, confirming its strong ability to induce cell death. We also monitored the translocation of phosphatidylserine (PS) to the outer surface of the cell membrane, which is generally considered as the hallmark of apoptosis.^28^ The fraction of Annexin V+/7-AAD-cells for the control was 1.73%, whereas those of complex **7**, complex **13**, and cisplatin were 11.4%, 25.0%, and 43.2%, respectively. To further investigate the ability of the complexes to induce apoptosis, the cells exposed to cisplatin or ruthenium compounds were stained with Hoechst 33342 to monitor any nuclear morphological change (Fig. 2A). Cisplatin and complex **7**-treated cells show characteristic apoptotic cell morphological changes including nuclear shrinkage and DNA fragmentation, which are consistent with the results from the cell cycle analysis and Annexin V/7-AAD double staining assay. The cells treated with complex **13**, however, lack any typical apoptotic morphology. Together with our observation that complex **13** does not induce cell cycle arrest, this ruthenium(iii) amidine complex may kill cancer cells through non-apoptotic cell death pathways. To further address the different mode of action between complexes **7** and **13**, we monitored the expression levels of DNA damage marker γ-H2AX upon treatment with the ruthenium complexes or cisplatin (Fig. 2B). Complex **7**- or cisplatin-treated cells displayed increased levels of γ-H2AX compared to the control group, which is consistent with their morphological features of apoptosis that we have observed. In contrast, complex **13** induced no change over 48 h, indicating that complex **13** is not a DNA-damaging agent.

**Fig. 3:**
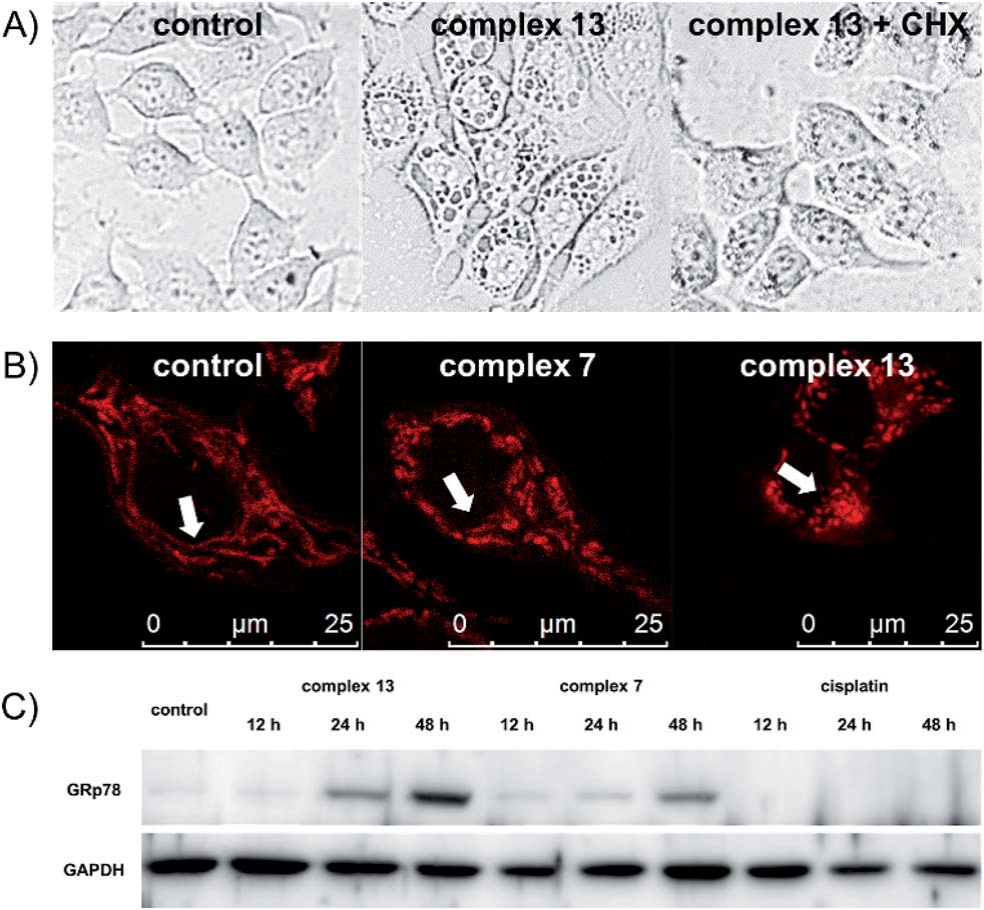
(A) Confocal images of MCF-7 cells treated with 3 μM complex **13** for 4 h with or without the pretreatment of inhibitors. (B) Morphology of mitochondria in MCF-7 cells upon treatment with 0.5 μM complex **7**, or 0.1 μM complex **13**. (C) Western blot analysis of ER stress marker GRp78 in MCF-7 cells upon treatment with complex **13**, complex **7**, or cisplatin at their IC_70_ values for different periods of time.

One remarkable phenomenon that we observed in the Hoechst-staining assay is the formation of massive vacuoles and a lack of apoptotic morphology in MCF-7 cells treated with complex **13** but not with complex **7** (Fig. 2), which are the hallmarks of autophagy or paraptosis.^29–32^ We first treated the cells with cycloheximide (CHX), a protein synthesis inhibitor, and then exposed the cells to complex **13**. The formation of vacuoles is apparently reduced by the pretreatment of CHX (Fig. 3A), indicating that protein synthesis is required for the complex **13**-induced cell death process. We subsequently investigated if complex **13** triggers autophagic cell death pathways. During the autophagy process, a multilamellar spherical structure called an autophagosome is formed. We introduced dansylcadaverine (MDC), a fluorescent probe that is incorporated into multi-membrane bodies, to label the autophagosome.^33^ The cells exposed to tamoxifen, a positive control,^34^ show stronger MDC-labeled signals compared to the untreated group, indicating the formation of autophagosomes. Conversely, the cells treated with complex **13** show an identical level of MDC accumulation compared to the untreated cells (Fig. S7†), indicating that complex **13**-induced cytoplasmic vacuoles cannot be labeled by MDC and thus these vacuoles are not autophagosomes. In addition, the formation of autophagosomes highly relies on the activation of class III phosphoinositide 3-kinases (PI3K). MCF-7 cells were pre-treated with wortmannin, a PI3K inhibitor,^35^ and then treated with complex **13**. This ruthenium(iii) amidine complex is still able to induce vacuoles in wortmannin pre-treated cells, confirming that the formation of vacuoles is not an autophagy process (Fig. S8A†). Together with the result from the MDC treatment, the possibility of autophagy as the cell death pathway for complex **13** is therefore excluded.

Finally, we tested if paraptosis is the major cell death mode for cells treated with complex **13**. The dilation of the mitochondria is commonly reported during paraptotic processes.^29^ We monitored mitochondrial morphology changes upon treatment by staining MCF-7 cells with MitoTracker Red. Mitochondria in the control and tamoxifen-treated groups remained as the normal fiber-like structures. In contrast, upon treatment with complex **13** but not **7**, the mitochondria appeared as spherical (Fig. 3B and S8B†), indicating the mitochondria dilation.^36^ During paraptosis cell death, endoplasmic reticulum (ER) stress is also commonly reported.^29^ Along with the observation that complex **13** mainly localizes in the cytoplasm after entering cells, we further investigated the ability of ruthenium compounds to induce ER stress. The expression level of the ER stress marker GRp78 was monitored. Complex **13**-treated cells show significant upregulation of GRp78 compared to the untreated or complex **7**-treated cells, confirming the ability of complex **13** to induce paraptosis. Cisplatin, however, does not induce ER stress in 48 h (Fig. 3C).

It is worth noting that complex **13** also induces PS translocation as revealed above, which is a general apoptosis feature. This observation does not conflict with our conclusion that complex **13** induces paraptosis-like cell death, because the exposure of PS in a paraptosis-like cell death process has been demonstrated previously.^37^ Cells treated with complex **13** also lack typical apoptotic morphology, characterized by massive vacuole formation, mitochondrial swelling, and ER stress. Therefore, complex **13** induces non-apoptosis and non-autophagy cell death pathways with properties meeting the descriptions of paraptosis.

## Conclusions

**Fig. 4:**
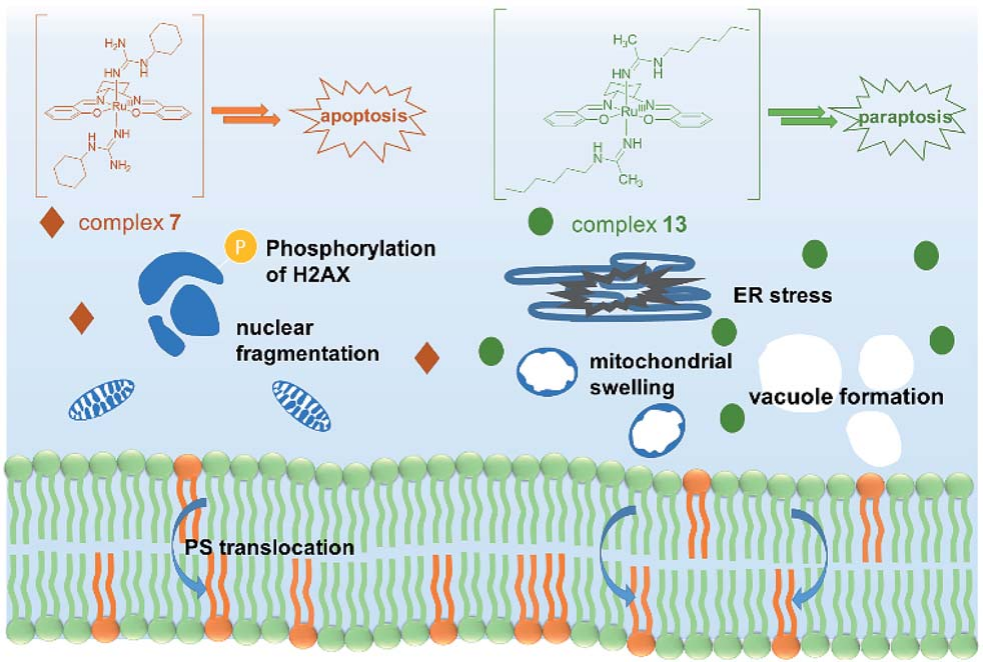
Proposed modes of action of complexes **7** and **13**. Complex **7** leads to an apoptosis pathway, whereas complex **13** induces paraptosis-like cell death.

In conclusion, two novel series of bis(guanidine) and bis(amidine)ruthenium(iii) complexes were obtained and were well characterized. The leading complexes, which are stable in aqueous solution (Fig. S2†), effectively kill cancer cells but not normal cells. The bis(guanidine)ruthenium(iii) complex **7** induces apoptosis to kill cancer cells (Fig. 4). In stark contrast, the bis(amidine)ruthenium(iii) complex **13** does not show strong evidence of DNA binding, cell cycle arrest, nor apoptotic morphology, but induces paraptosis, as evidenced by massive vacuole formation, mitochondrial dilation, and ER stress. Thus, we provide the first example of (salen)ruthenium(iii) anticancer agents that exhibit distinct modes of cell death directed by guanidine or amidine axial ligands. Our study guides the design of (salen)ruthenium(iii) complexes as a new class of ruthenium-based anticancer drug candidate that has completely different mechanisms to cisplatin. The detailed mechanism of the significant cancer-cell selectivity of complex **7** is still under investigation.
