# Supplementary material for: Cytotoxic (salen)ruthenium(iii) anticancer complexes exhibit different modes of cell death directed by axial ligands
Source: Chem Sci. 2017 Jul 31;8(10):6865–70. doi: 10.1039/c7sc02205k (PMC5632802; doi:10.1039/c7sc02205k)
Supplement: Supplementary file 1 [file SC-008-C7SC02205K-s001.pdf]

Electronic Supplementary Material (ESI) for Chemical Science.
This journal is © The Royal Society of Chemistry 2017

Supporting Information

Cytotoxic (Salen)ruthenium(III) Anticancer Complexes Exhibit Different Modes of Cell Death Directed by Axial Ligands

Experimental section---P 1-8
Supplementary Figures & Tables---P 9-21

Experiment

Materials

$[Ru^{III}(L)(H_2O)_2](PF_6)$ and $[Ru^{VI}(N)(L)(MeOH)](PF_6)$ were prepared according to literature procedures.[1-2] The Schiff base ligand, H_2L (L = *N*,*N*'-bis(salicylidene)-*o*-cyclohexylenediamine dianion) was synthesized by condensation of salicylaldehyde with *trans*-1,2-cyclohexyldiamine in refluxing ethanol. Acetonitrile used for electrochemistry were distilled over calcium hydride. All other chemicals were of reagent grade and used without further purification. All manipulations were performed without precaution to exclude air or moisture unless otherwise stated.

Physical Measurements

IR spectra were obtained as KBr discs using a Nicolet 360 FT-IR spectrophotometer. Elemental analysis was performed using an Elementar Vario EL Analyzer. Magnetic measurements were performed at room temperature using a Sherwood magnetic balance (Mark II). Electrospray ionization mass spectrometry (ESI-MS) was performed with a PE-SCIEX API 2000 triple quadruple mass spectrometer. Cyclic voltammetry was performed with a PAR model 273 potentiostat using a glassy carbon working electrode, a $Ag/AgNO_3$ (0.1 M in CH_3CN) reference electrode, and a Pt wire counter electrode with ferrocene as the internal standard.

X-ray Crystallography

Diffraction data of complexes **3** and **15** were collected with an Oxford Xcalibur, Sapphire 3, Gemini ultra-diffractometer using graphite-monochromated Cu Kα radiation (λ=1.54178Å) at 193 K. The collected frames were processed with the software CrystAlisPro. The structures were solved by direct method (SHELXTL) in conjugation with standard difference Fourier technique and subsequently refined by full matrix least-squares analyses on F^2.[3-4] Hydrogen atoms were generated in their idealized position and all non-hydrogen atoms were assigned with isotopic displacement parameters.[5]

Synthesis

***Trans*-[Ru^{III}(L)(N≡CNH_2)$_2$]PF_6 (2)** Solid NH_2CN (126 mg, 3 mmol) was added to [Ru^{III}(L)(H_2O)$_2$]PF_6 (180 mg, 0.3 mmol) in 10 mL THF. The mixture was heated to reflux for 2 h, cooled to room temperature and concentrated to ca. 1 mL. Addition of diethyl ether (20 mL) gave a green solid which was filtered and washed with diethyl ether. The solid was recrystallized from dry THF. Yield: 88 mg, 0.135 mmol, 45%. IR (KBr, cm^{-1}): ν(N–H) 3378, 3320; ν(C≡N) 2276, ν(P–F) 845. Anal. Calcd. for $C_{22}H_{24}N_6O_2PF_6Ru$: C, 40.62; H, 3.72; N, 12.92. Found: C, 40.41; H, 3.86; N, 12.69. ESI-MS: m/z 506 (M^+). Magnetic susceptibility: μ_{eff} = 1.96 μB.

***Trans*-{Ru^{III}(L)[NH=C(NH_2)$_2$]$_2$}PF_6 (3)** NH_3 gas was bubbled into a solution of **2** (50 mg, 0.077 mmol) in THF (15 mL) with heating (~50 °C) for 1 h. Then the reaction mixture was cooled to room temperature, filtered and evaporated to dryness under vacuum. The residue was re-dissolved in minimum amount of CH_2Cl_2 and loaded onto a neutral alumina column. The purple layer eluted by MeOH/CH_2Cl_2 (1:20, *v/v*) was evaporated to dryness to give **3** as a purple solid. Slow diffusion of diethyl ether into the methanolic solution of **3** resulted in the formation of purple single crystals. Yield: 9.5 mg, 0.014 mmol, 18%. IR (KBr, cm^{-1}): ν(N-H) 3465, 3410, 3372, 3217; ν(C=N) 1647; ν(P-F) 845. Anal. Calcd. for $C_{22}H_{30}N_8O_2PF_6Ru$: C, 38.60; H, 4.42; N, 16.37. Found: C, 38.72; H, 4.54; N, 16.22. ESI-MS: m/z 540 (M^+). Magnetic susceptibility: μ_{eff} = 1.98 μ_B.

***Trans*-{Ru^{III}(L)[NH=C(NH_2)(NHC_3H_7)]$_2$}PF_6 (4)** Propylamine (82 μL, 1.00 mmol) was added to **2** (50 mg, 0.077 mmol) in THF (15 mL) and the mixture was heated to reflux for 5 h. The dark green solution was cooled to room temperature and then concentrated to *ca*. 1 mL. Diethyl ether (40 mL) was added to precipitate a solid. The crude product was re-dissolved in minimum amount of CH_2Cl_2, loaded onto a neutral alumina column and eluted by MeOH/CH_2Cl_2 (1:20, *v/v*). The second purple band was collected and evaporated to dryness to give **4** as a purple solid. Yield: 14.8 mg, 0.019 mmol, 25%. IR (KBr, cm^{-1}): ν(N-H) 3476, 3412, 3265; ν(C=N) 1645; ν(P-F) 845. Anal. Calcd. for $C_{28}H_{42}N_8O_2PF_6Ru$: C, 43.75; H, 5.51;

N, 14.58. Found: C, 43.61; H, 5.74; N, 14.38. ESI-MS: m/z 624 (M^+). Magnetic susceptibility: $\mu_{eff} = 1.88\ \mu_B$.

***Trans*-{Ru^{III}(L)[NH=C(NH_2)(NHC_4H_9)]$_2$}PF_6 (5)** The preparation is similar to that of **4** except using butylamine (100 μL, 1.00 mmol) instead of propylamine. Yield: 12.8 mg, 0.016 mmol, 21%. IR (KBr, cm^{-1}): ν(N-H) 3460, 3420, 3265; ν(C=N) 1642; ν(P-F) 845. Anal. Calcd. for $C_{30}H_{46}N_8O_2PF_6Ru$: C, 45.22; H, 5.82; N, 14.06; Found: C, 45.11; H, 6.00; N, 13.78. ESI-MS: m/z 652 (M^+). Magnetic susceptibility: $\mu_{eff} = 2.09\ \mu_B$.

***Trans*-{Ru^{III}(L)[NH=C(NH_2)(NHCH(CH_3)$_2$)]$_2$}PF_6 (6)** Isopropylamine (82 μL, 1.00 mmol) was added to **2** (50 mg, 0.077 mmol) in THF (15 mL) and the mixture was refluxed for 6 h. Solids of **6** were gradually formed from the reaction mixture upon cooling to room temperature. The solid was filtered, washed with diethyl ether and air dried. Recrystallization from MeOH/Et_2O gave **6** as purple crystals. Yield: 20.7 mg, 0.027 mmol, 35%. IR (KBr, cm^{-1}): ν(N-H) 3494, 3402, 3365; ν(C=N) 1637, 1599; ν(P-F) 843. Anal. Calcd. for $C_{28}H_{42}N_8O_2PF_6Ru$: C, 43.75; H, 5.51; N, 14.58. Found: C, 43.61; H, 5.74; N, 14.38. ESI-MS: m/z 624 (M^+). Magnetic susceptibility: $\mu_{eff} = 1.93\ \mu_B$.

***Trans*-{Ru^{III}(L)[NH=C(NH_2)(NHC_6H_{11})]$_2$}PF_6 (7)** The preparation is similar to that of **6** except using cyclohexylamine (115 μL, 1.00 mmol) instead of isopropylamine. Yield: 14.4 mg, 0.017 mmol, 22%. IR (KBr, cm^{-1}): ν(N-H) 3466, 3404; ν(C=N) 1639, 1606; ν(P-F) 847. Anal. Calcd. for $C_{34}H_{50}N_8O_2PF_6Ru$: C, 48.11; H, 5.94; N, 13.20; Found: C, 47.93; H, 5.88; N, 13.32. ESI-MS: m/z 704 (M^+). Magnetic susceptibility: $\mu_{eff} = 1.94\ \mu_B$. The log P value is 1.67 ± 0.08.

***Trans*-{Ru^{III}(L)[NH=C(NH_2)(NHC_2H_4OH)]$_2$}PF_6 (8)** The preparation is similar to that of **4** except using ethanolamine (60 μL, 1.00 mmol) instead of propylamine. Yield: 14.9 mg, 0.019 mmol, 25%. IR (KBr, cm^{-1}): ν(O-H) 3473; ν(N-H) 3268; ν(C=N) 1640; ν(P-F) 847. Anal. Calcd. for $C_{26}H_{38}N_8O_4PF_6Ru$: C, 40.42; H, 4.96; N, 14.50; Found: C, 40.57; H, 4.68; N, 14.28. ESI-MS: m/z 628 (M^+). Magnetic susceptibility: $\mu_{eff} = 2.03\ \mu_B$.

***Trans*-[Ru^{III}(L)(N≡CCH_3)$_2$]PF_6 (9)** [Ru^{VI}(L)(N)(MeOH)]PF_6 (200 mg, 0.327 mmol) was dissolved in CH_3CN (100 mL) and refluxed under argon overnight. The resultant solution was cooled to room temperature and then filtered. The filtrate was evaporated to dryness under reduced pressure to give green solids of **9**. Yield: 183 mg, 0.28 mmol, 85%. IR (KBr, cm^{-1}): ν(C=N) 1602; ν(P-F) 841. Anal. Calcd. for $C_{24}H_{26}N_4O_2PF_6Ru$: C, 44.45; H, 4.04; N, 8.64; Found: C, 44.37; H, 4.18; N, 8.82. ESI-MS: m/z 504 (M^+). Magnetic susceptibility: $\mu_{eff} = 1.88\ \mu_B$.

***Trans*-{Ru^{III}(L)[NH=C(CH$_3$)(NHC$_3$H$_7$)]$_2$}PF$_6$ (10)** Propylamine (82 μL, 1.00 mmol) was added to **9** (100 mg, 0.154 mmol) in THF (30 mL) and the mixture was refluxed for 5 h. The resultant dark green solution was then concentrated to *ca*. 1 mL. Addition of diethyl ether (40 mL) resulted a green solid. The solid was dissolved in minimum amount of CH_2Cl_2, loaded onto a neutral alumina column and eluted by MeOH/ CH_2Cl_2 (1: 20, *v/v*). The second green band was collected and evaporated to dryness to give **10** as a green solid. Yield: 29.5 mg, 0.038 mmol, 25%. IR (KBr, cm^{-1}): ν(N-H) 3364, 3268; ν(C=N) 1635, 1600; ν(P-F) 841. Anal. Calcd. for $C_{30}H_{44}N_6O_2PF_6Ru$: C, 46.99; H, 5.78; N, 10.96; Found: C, 46.82; H, 5.97; N, 10.68. ESI-MS: m/z 622 (M^+). Magnetic susceptibility: $\mu_{eff} = 1.94\ \mu_B$.

***Trans*-{Ru^{III}(L)[NH=C(CH$_3$)(NHC$_4$H$_9$)]$_2$}PF$_6$ (11)** The preparation is similar to that of **10** except using n-butylamine (100 μL, 1.00 mmol) instead of propylamine. Yield: 34.3 mg, 0.043 mmol, 28%. IR (KBr, cm^{-1}): ν(N-H) 3364, 3268; ν(C=N) 1635, 1601; ν(P-F) 842. Anal. Calcd. for $C_{32}H_{48}N_6O_2PF_6Ru$: C, 48.36; H, 6.09; N, 10.57; Found: C, 48.49; H, 6.21; N, 10.59. ESI-MS: m/z 650 (M^+). Magnetic susceptibility: $\mu_{eff} = 1.89\ \mu_B$.

***Trans*-{Ru^{III}(L)[NH=C(CH$_3$)(NHCH$_2$CH$_2$NH$_2$)]$_2$}PF$_6$ (12)** The preparation is similar to that of **10** except using ethylenediamine (67 μL, 1.00 mmol) instead of propylamine. Yield: 26 mg, 0.033 mmol, 22%. IR (KBr, cm^{-1}): ν(N-H) 3362, 3263, 3202; ν(C=N) 1632, 1600; ν(P-F) 841. Anal. Calcd. for $C_{28}H_{42}N_8O_2PF_6Ru$: C, 43.75; H, 5.51; N, 14.58; Found: C, 43.55; H, 5.54; N, 14.81. ESI-MS: m/z 624 (M^+). Magnetic susceptibility: $\mu_{eff} = 1.99\ \mu_B$.

***Trans*-{Ru^{III}(L)[NH=C(CH$_3$)(NHC$_6$H$_{13}$)]$_2$}PF$_6$ (13)** The preparation is similar to that of **10** except using n-hexylamine (131 μL, 1.00 mmol) instead of propylamine. Yield: 36.6 mg, 0.043 mmol, 28%. IR (KBr, cm^{-1}): ν(N-H) 3364, 3269; ν(C=N) 1635, 1600; ν(P-F) 841. Anal. Calcd. for $C_{36}H_{56}N_6O_2PF_6Ru$: C, 50.82; H, 6.63; N, 9.88; Found: C, 50.57; H, 6.71; N, 9.69. ESI-MS: m/z 706 (M^+). Magnetic susceptibility: $\mu_{eff} = 1.90\ \mu_B$. The log P value is 1.91 ± 0.12.

***Trans*-{Ru^{III}(L)[NH=C(CH$_3$)(NC$_4$H$_8$O)]$_2$}PF$_6$ (14)** The preparation is similar to that of **10** except using morpholine (87 μL, 1.00 mmol) instead of propylamine. Yield: 31.7 mg, 0.038 mmol, 25%. IR (KBr, cm^{-1}): ν(N-H) 3634, 3346; ν(C=N) 1600, 1558; ν(P-F) 839. Anal. Calcd. for $C_{32}H_{44}N_6O_4PF_6Ru$: C, 46.71; H, 5.39; N, 10.21; Found: C, 46.92; H, 5.62; N, 9.97. ESI-MS: m/z 678 (M^+). Magnetic susceptibility: $\mu_{eff} = 1.96\ \mu_B$.

***Trans*-{Ru^{III}(L)[NH=C(NH$_2$)(NHC$_2$H$_4$OH)]$_2$}PF$_6$ (15)** The preparation of is similar to that of **10** except using ethanolamine (60 μL, 1.00 mmol) instead of propylamine. Slow diffusion of diethyl ether into a methanolic solution of **15** resulted in the formation of dark single

crystals. Yield: 38 mg, 0.049 mmol, 32%. IR (KBr, cm^{-1}): ν(N-H) 3612, 3362, 3208; ν(O-H) 3287; ν(C=N) 1634, 1599; ν(P-F) 842. Anal. Calcd. for $C_{28}H_{40}N_6O_4PF_6Ru$: C, 43.64; H, 5.23; N, 10.90; Found: C, 43.39; H, 5.28; N, 11.19. ESI-MS: m/z 626 (M^+). Magnetic susceptibility: $\mu_{eff} = 1.94\ \mu_B$.

Cell lines and cell culture conditions

Human epithelial carcinoma HeLa cells, human lung carcinoma A549 cells, human breast carcinoma MCF-7 cells, and human liver carcinoma HepG2 cells were cultured in DMEM with 10% FBS and 100 units penicillin/streptomycin. Human fetal lung fibroblast MRC-5 and WI-38 cells were cultured in MEM with 10% FBS, 1% L-glutamine, 100 units penicillin/streptomycin, and 1% sodium pyruvate. Human ovarian carcinoma A2780 and cisplatin-resistant A2780cisR cells were cultured in RPMI-1640 with 10% FBS, 100 units penicillin/streptomycin, and 1% L-glutamine. 5 μM of cisplatin was added into culture medium for A2780cisR to maintain the resistance. All the cells were cultured at 37 ºC in 5% CO_2.

3-(4,5-dimethylthiazol-2-yl)-2,5-diphenyltetrazolium bromide (MTT) assay

The cytotoxicity profile of Ru complexes against selected cell lines was obtained by MTT assay. Cisplatin was introduced as control. For instance, MCF-7 cells were seeded into 96-well plates at a density of 2000 cells per well and incubated for 24 h. The complexes were dissolved in DMF to make the stock solution. Certain amount of the stock solution was digested with 65% HNO_3 at 60°C overnight and the metal level was determined by ICP-OES. Stock solution of complex was diluted into culture medium with various final concentration. Cells were then treated with medium containing various concentrations of compounds for 72 h at 37 ºC in 5% CO_2. DMF was used to increase the solubility of compounds, and the final concentration of DMF was 1% (v/v). After the drug treatment for 72 h, the compound-containing medium was replaced by FBS-free medium with 1 mg/mL MTT for 2 h incubation. DMSO was added to each well when the medium containing MTT was removed after incubation. The absorbance was measured at 570 and 630 nm. Cells incubated with medium containing 1% DMF only were used as controls.

Stability of complexes 7 and 13

The stability of ruthenium compounds was tested by UV-Vis spectrophotometer (Shimadzu UV-1700). The ruthenium compound solution in DMF/PBS (v/v=1/1) was prepared at the

concentration of 50 μM (for complex **13**) or 20 μM (for complex **7**) and incubated at 37 ºC. To test the absorption pattern of reduced ruthenium compound, access amount of ascorbic acid was added into the system and incubated at 37 ºC. For the stability in medium, the ruthenium compound solution in phenol red free DMEM medium (10% FBS, 100 units penicillin/streptomycin, 10% DMF) was prepared at the concentration of 20 μM. The corresponding solvent was used as background test. At certain time points the UV-Vis absorption spectra were recorded.

Measurement of water-octanol partition coefficient (log P)

The ruthenium compound was dissolved in octanol. 1 mL of solution was incubated with 1 mL of PBS buffer at 23 ºC overnight. The two phases were separated by centrifugation and then digested with 65% HNO_3 at 60℃overnight. The Ru level was determined by ICP-MS (PE Elan 6100 DRC). The octanol used in this assay was pre-saturated with PBS buffer.

Measurement of aqueous solubility

Excess amount of ruthenium complex in MilliQ was incubated under dark at room temperature overnight. The mixture centrifuged and the Ru level in the supernatant was measured by ICP-OES. The aqueous solubility of complex **7** and **13** is 4.06±0.89 and 1.62±0.56 μM, respectively.

Calf thymus DNA (CT-DNA) binding

0.15 mg of CT-DNA was incubated with varying concentrations of cisplatin, complex **7**, or **13** in a binding buffer (12 mM K_3PO_4, 140 mM $NaClO_4$, pH 7.4) at 37 ºC overnight. The metal-DNA adduct was then precipitated by ethanol. The pellets were washed with 70% ethanol for 3 times, vacuum dried, and then re-dissolved in a Tris-HCl buffer (10 mM, pH 7.4). The DNA and metal concentrations were determined by nanodrop 1000 spectrophotometer and ICP-OES (PE 2100DV), respectively.

Plasmid DNA interaction

eGFP-N1 plasmid DNA was dissolved in a binding buffer (12 mM K_3PO_4, 140 mM $NaClO_4$, pH 7.4) and the concentration was determined by nanodrop spectrophotometer. 200 ng of plasmid DNA was incubated with cisplatin or ruthenium complex at metal/base pair molar

ratio of 0.5/1, 1/1, or 5/1. After 8 h, the DNA solution was mixed with loading buffer (10 mM Tris, 0.03% bromophenol blue, 0.03% xylene cyanol FF, 60% glycerol, 60 mM EDTA, pH 7.6) and separated on 1% agarose gel at 75 V for 60 min. The gel was stained with 1% GelRed and visualized on a Bio-Rad ChemiDoc Touch System.

Cellular accumulation of Ru

MCF-7 cells were plated into 6-well plates and treated with 10 μM Ru complex for 8 h. Cells were washed with ice-cold PBS for 3 times and collected by trypsinization. The number of cells was determined by a hemacytometer. The Ru level was measured by ICP-OES after the digestion of cell pellets with 65% HNO_3 at 60℃ overnight.

Ru distribution in cytoplasm, nucleus, and membrane

MCF-7 cells were treated with 10 μM Ru complex for 8 h. After washing for 3 times, cells were collected by trypsinization and cell number was determined by a hemacytometer. Isolation of cytoplasm, nucleus, and membrane was performed following the protocol of a Nuclear and Cytoplasmic Extraction Kit (Thermo Scientific 78835). The fractions were digested with 65% HNO_3 at 60℃ overnight. Ru level was determined by ICP-MS (PE Elan 6100 DRC).

Accumulation on genomic DNA

MCF-7 cells were plated in 10-am dishes. Cells were treated with 10 μM Ru complex or cisplatin for 8 h, followed by washing with ice-cold PBS for 3 times. The cells were then scraped into PBS buffer and the cell number was determined by a hemacytometer. The cells were centrifuged and resuspended in lysis buffer (100 mM Tris, 5 mM EDTA, 0.2% SDS, 200 mM NaCl, 100 μg/mL proteinase K, pH 8.5). The cell lysate was incubated at 55℃ overnight, extracted with phenol/chloroform/isoamyl alcohol for twice the chloroform for twice. Genomic DNA was precipitated with 0.7x isopropanol, washed twice with cold 70% ethanol, and dissolved in Tris buffer (10 mM Tris, 1 mM EDTA, 50 μg/mL RNase A). The concentration of DNA and metal was determined by nanodrop and ICP-OES, respectively.

Cell cycle analysis

MCF-7 cells were seeded into 6-well plates and incubated for 24 h. Fresh medium containing different concentrations of cisplatin or Ru complex was added. After 24 h, the cells were washed with PBS for 3 times and collected by trypsinization. The cells were subsequently washed with PBS for another time and fixed with 70% ethanol at 4 ºC overnight. Finally, the cells were washed with PBS, suspended in a PI solution (0.1% Triton X-100, 200 μg/mL RNase A, 20 μg/mL PI in PBS), and stained for 15 min at 37 ºC. The cell cycle distribution was examined by flow cytometry.

Hoechst staining assay

MCF-7 cells were seeded into 24-well plates and allowed to attach for 24 h. Medium containing 30 μM of cisplatin or Ru complex was added to cells. After 24 h treatment, cells were stained with Hoechst (5 μg/mL in PBS) at 37 °C for 20 min, followed by washing three times with PBS. Fluorescent samples were recorded on a laser confocal scanning microscope (Leica SPE).

Apoptosis assay

MCF-7 cells were seeded into 6-well plates and allowed to attach for 24 h. Medium containing different concentrations of cisplatin or Ru complex was added to the cells. After 24 h treatment, the cells were collected by trypsinization and washed with ice-cold PBS and then with annexin V-binding buffer (10 mM HEPES, 140 mM NaCl, 2.5 mM $CaCl_2$, pH 7.4). Samples were staining with 5 μL annexin-V and 1 μL PI (100 μg/mL) at r.t. for 15 min. An FACS Calibur flow cytometer was utilized for the analysis.

Dansylcadaverine (MDC) staining assay

MCF-7 cells were seeded into 24-well plates and allowed to attach for 24 h. Medium containing 10 μM of tamoxifen or 0.1 μM of complex **13** was added to the cells. After 24 h treatment, the cells were stained with MDC (50 μM in PBS) at 37 °C for 10 min, followed by washing three times with PBS. Fluorescent samples were recorded on a laser confocal scanning microscope (Leica SPE).

Vacuolization inhibition

MCF-7 cells were seeded into 24-well plates and allowed to attach for 24 h. Cells were then pretreated with 10 μM of wortmannin, 60 μM of necrostatin-1 for 4 h or 20 μM of

cycloheximide (CHX) overnight. The growth medium was removed and the cells were exposed to 9 μM of complex **7** or 3 μM of complex **13**. After 4 h treatment, the cells were washed three times with PBS and observed under a laser confocal scanning microscope (Leica SPE).

Mitochondrial swelling assay

MCF-7 cells were seeded into 15 mm glass bottom dishes and allowed to attach for 24 h. Medium containing 10 μM of tamoxifen, 0.5 μM of complex **7**, or 0.1 μM of complex **13** was added to cells. After 24 h treatment, cells were stained with MitoTracker Red (0.1 μM in PBS) at 37 °C for 20 min, followed by washing three times with PBS. Fluorescent samples were recorded on a laser confocal scanning microscope (Leica SP5).

Western blot

MCF-7 cells were seeded into 6-well plates and incubated for 24 h. Cells were then treated with cisplatin or ruthenium compounds at their IC70 values. After incubation for 12, 24, 48 h, cells were washed 3 times with ice-cold PBS and then treated with western lysis buffer (Beyotime P0013) compensated with proteinase inhibitor (1 mM PMSF and protease inhibitor cocktail, Roche) for 30 min on ice. The cells lysates were collected and centrifuged. The protein level in the supernatant was determined by BCA assay (Beyotime P0012). Fixed amount of protein samples were mixed with Laemmli buffer (0.05 M Tris-Cl, 0.1 M dithiothreitol, 10% glycerol, 2% SDS, 0.05% bromophenol blue, pH 6.8) and denatured at 95°C for 5 min, separated on SDS-PAGE gel and transferred to PVDF membrane (Millipore). The membrane was incubated with GRp78 (SantaCruz, 1:1000), GAPDH (SantaCruz, 1:10000), γ-H2AX (Cell Signaling, 1:1000), and β−actin (Cell Signaling, 1:2000) primary antibodies and then a secondary antibody (Goat-anti-rabbit/rabbit-anti-mouse, SantaCruz). Protein bands were visualized chemiluminescence detection system (Millipore).

Supplementary Figures & Tables

Table S1. Electrochemical and IR (KBr) data for complexes **3–8** and **10–15**. [a]Glassy carbon working electrode, Pt counter electrode, $Ag/AgNO_3$ reference electrode, 0.1 M $[^{n}Bu_4N]PF_6$ in CH_3CN as supporting electrode. Ferrocene was added as an internal standard. The ΔE values for the reversible couples in CH_3CN are 65-75 mV at a scan rate of 100 mVs^{-1}.

**Complexes**	**$E_{1/2}$ (V vs $Cp_2Fe^{+/0}$)[a]**		**IR (KBr, cm^{-1})**	
	**[$Ru^{III/II}$]**	**[$Ru^{IV/III}$]**	**ν(N-H)**	**ν(C=N)**
**3**	-1.21	+0.28	3465, 3410, 3372, 3217	1647
**4**	-1.23	+0.25	3476, 3412, 3265	1645
**5**	-1.26	+0.23	3460, 3420, 3265	1642
**6**	-1.32	+0.24	3494, 3402, 3365	1637, 1599
**7**	-1.26	+0.24	3466, 3404	1639, 1606
**8**	-1.18	+0.26	3268	1640
**10**	-1.13	+0.36	3364, 3268	1635, 1600
**11**	-1.13	+0.36	3364, 3268	1635, 1601
**12**	-1.09	+0.44	3362, 3263, 3202	1632, 1600
**13**	-1.13	+0.36	3364, 3269	1635, 1600
**14**	-0.87	+0.76	3634, 3346	1600, 1558
**15**	-1.02	+0.34	3612, 3362, 3208	1634, 1599

Table S2. Crystal data and structure refinement details for complexes **3•Et$_2$O** and **15**.

	**3•Et$_2$O**	**15**
Empirical Formula	$C_{26}H_{40}N_8O_3PF_6Ru$	$C_{28}H_{38}N_6O_4PF_6Ru$
Formula Weight, M_r	758.70	768.68
Crystal Type	Monoclinic	Monoclinic
Space Group	P21/n	P2/c
a/Å	14.1727(4)	9.20780(13)
b/Å	16.5751(4)	10.49143(18)
c/Å	14.5768(4)	16.4278(3)
β, deg	110.762(3)	96.1345(14)
V/ Å^3	3201.92(15)	1577.88(4)
Z	4	2
D_c/ gcm^{-3}	1.574	1.618
F(000)	1556	786
No. of reflns, collected	5703	3009
No. of obsd reflns, ($I>2\sigma(I)$)	4677	2969
R[a]	0.0531	0.0377
Rw[b]	0.1572	0.1012
GOF	0.997	1.042
No. of parameters	444	234

[a]$R = \Sigma||F_o|-|F_c||\Sigma|F_o|$, [b]$R = [\Sigma\omega(|F_o|-F_c|)^2/\Sigma\omega F_o]]^{1/2}$

Table S3. Selected bond lengths (Å) and angles (deg) of complex **3•Et$_2$O**.

Ru1–N1	1.983(3)	C21–N3	1.313(6)
Ru1–N2	1.990(4)	C21–N4	1.344 (7)
Ru1–N3	2.061(4)	C21–N5	1.333(7)
Ru1–N6	2.059(4)	C22–N6	1.302(6)
Ru1–O1	2.031(3)	C22–N7	1.346(6)
Ru1–O2	2.022(3)	C22–N8	1.341(7)
N3–Ru1–N6	178.6(2)	N6–C22–N7	121.9(5)
N3–C21–N4	121.9(5)	N6–C22–N8	121.3(5)
N3–C21–N5	121.5(5)	N7–C22–N8	116.8(4)
N4–C21–N5	116.5(6)		

Table S4. Selected bond lengths (Å) and angles (deg) of complex **15**.

Ru1 – N1	2.070(3)	C1 – N2	1.330(4)
Ru1 – N3	1.981(2)	C1 – C2	1.500(4)
Ru1 – O1	2.032(2)	N2 – C3	1.462(4)
N1 – C1	1.306(4)	C4 – O2	1.421(8)
N1 – Ru1 – O1	93.76(9)	Ru1 – N1 – C1	132.4(2)
N1 – Ru1 – O1i	88.63(9)	N1 – C1 – N2	120.6(3)
N1 – Ru1 – N3	86.88(10)	N1 – C1 – C2	120.3(3)
N1 – Ru1 – N3i	86.88(10)	C2 – C1 – N2	119.2(3)
N1 – Ru1 – N1i	176.65(13)	C1 – N2 – C3	126.3(3)

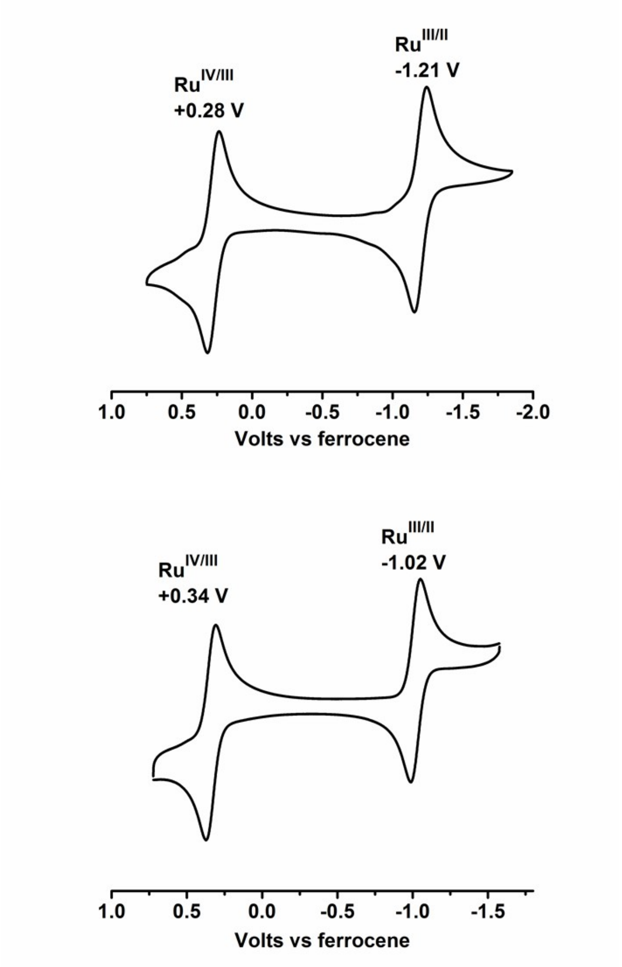

Figure S1. CVs of complexes **3** (top) and **15** (bottom) in CH_3CN.

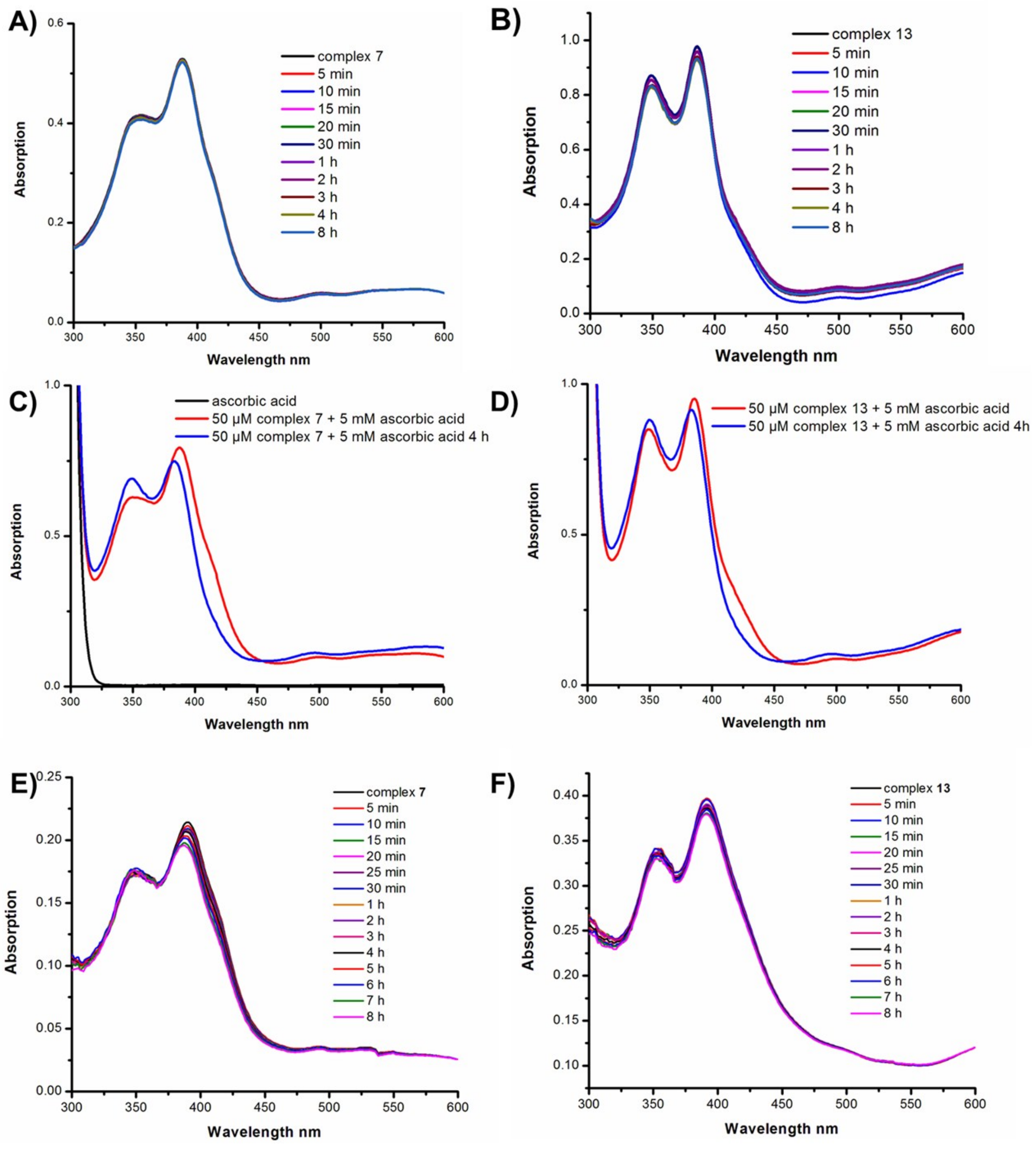

Figure S2. Stability of A) complex **7** in PBS/DMF (1: 1); B) complex **13** in PBS/DMF (1: 1); C) complex **7** incubated with excess amount of ascorbic acid; D) complex **13** incubated with excess amount of ascorbic acid; E) complex **7** in phenol red free DMEM (10% FBS, 10% DMF); F) complex **13** in phenol red free DMEM (10% FBS, 10% DMF), determined by UV-vis spectrophotometry.

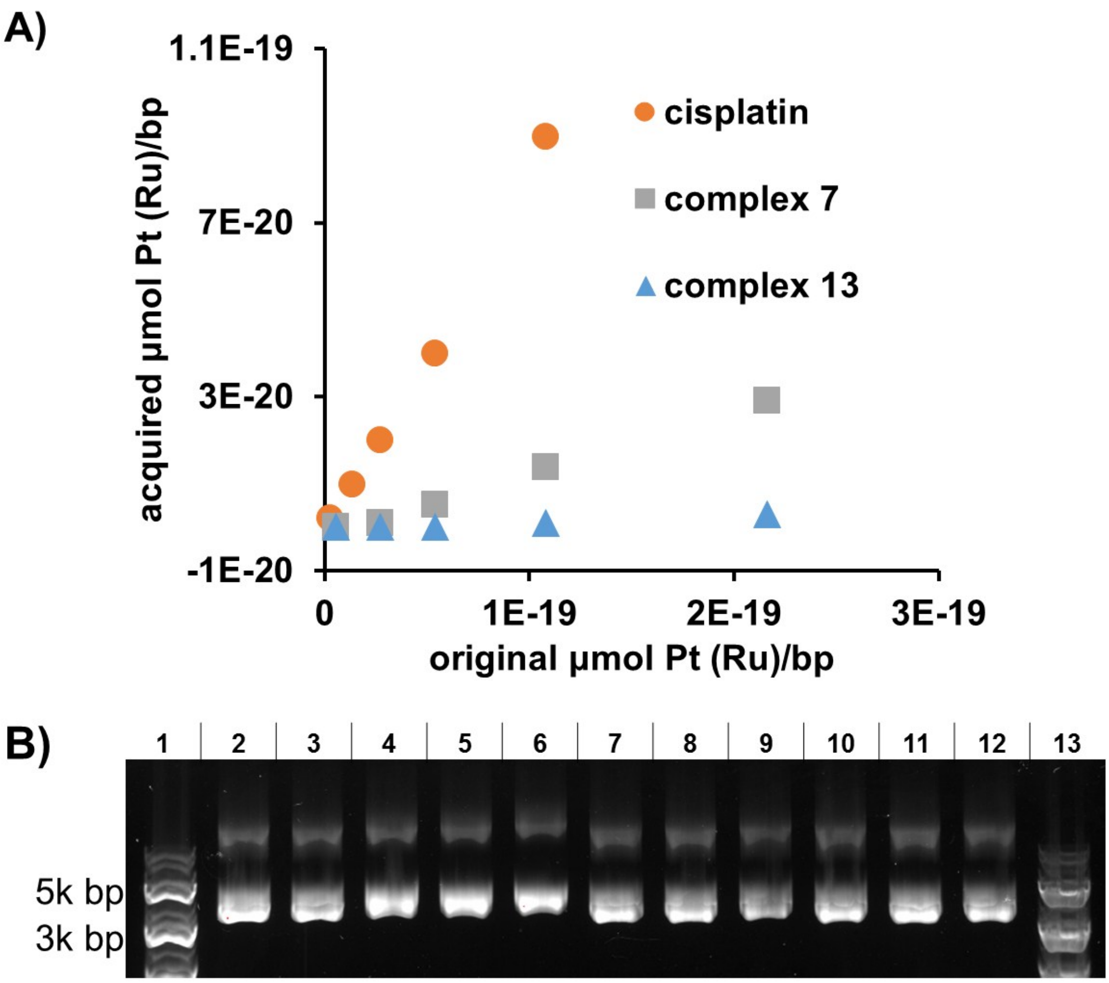

Figure S3. A) DNA binding of complex **7**, **13**, and cisplatin to Calf thymus DNA. B) eGFP plasmid DNA incubated with cisplatin or ruthenium complex for 8 h. Lane 1/13: DNA ladder; Lane 2: plasmid DNA; Lane 3: plasmid DNA+1%DMF; Lane 4/5/6: plasmid DNA+ cisplatin, molar ratio 0.5/1/5; Lane 7/8/9: plasmid DNA+ complex **7**, molar ratio 0.5/1/5; Lane 10/11/12: plasmid DNA+ complex **13**, molar ratio 0.5/1/5.

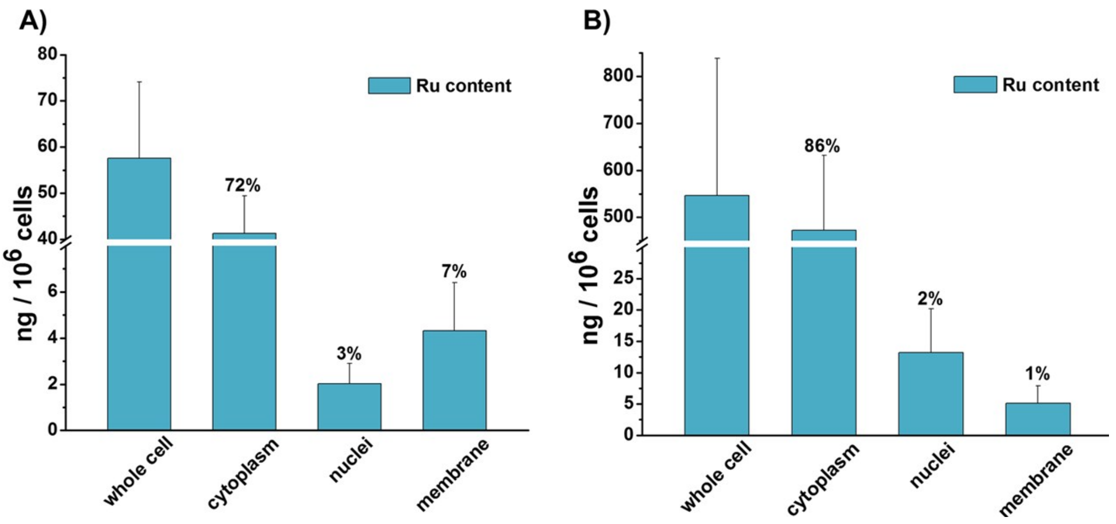

Figure S4. Ru accumulation levels of A) complex **7** and B) **13** in MCF-7 cells upon treatment with 10 μM complex for 8 h.

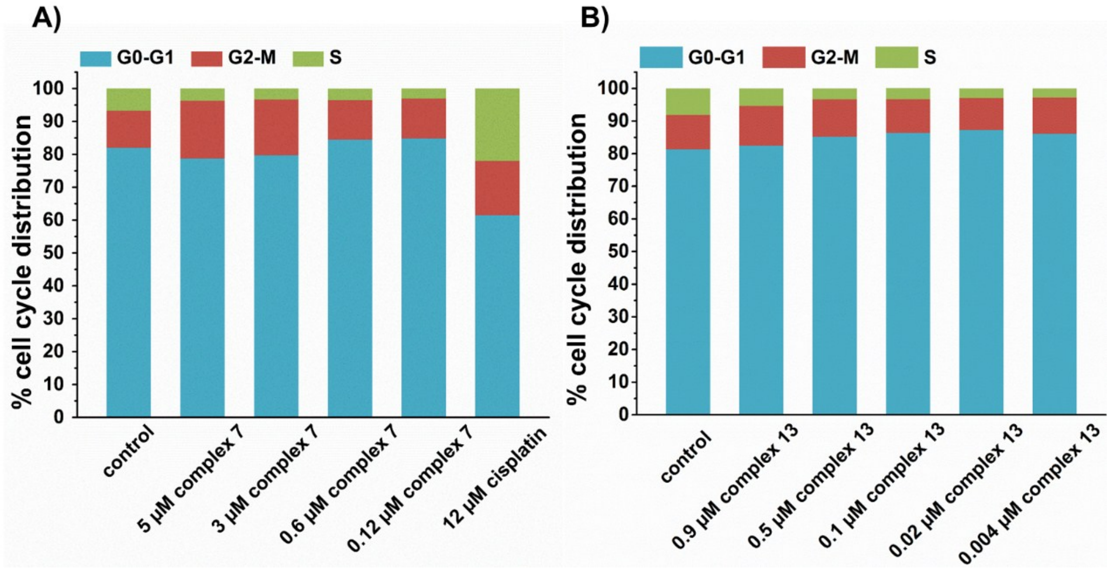

Figure S5. Cell cycle distribution of MCF-7 cells treated with A) complex **7** or B) **13** for 24 h.

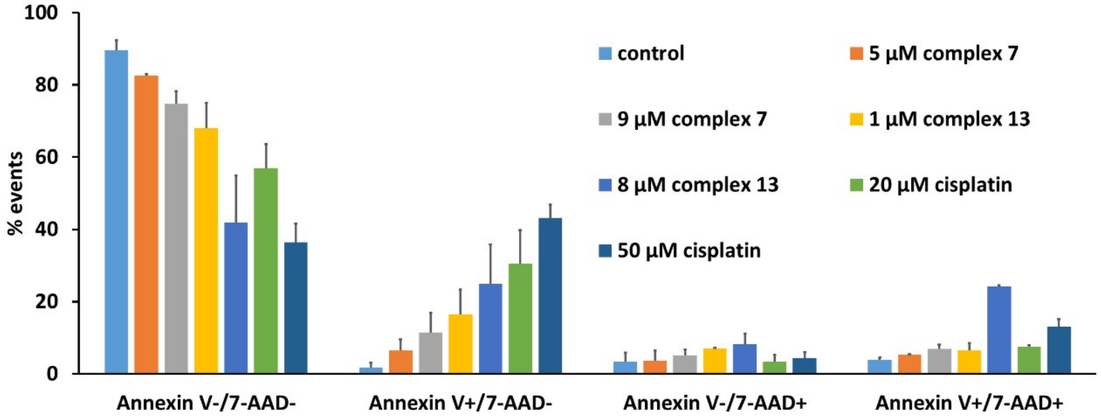

Figure S6. Annexin V/7-AAD double staining of MCF-7 cells exposed to cisplatin or ruthenium compounds at their IC_{70} or IC_{90} values for 24 h.

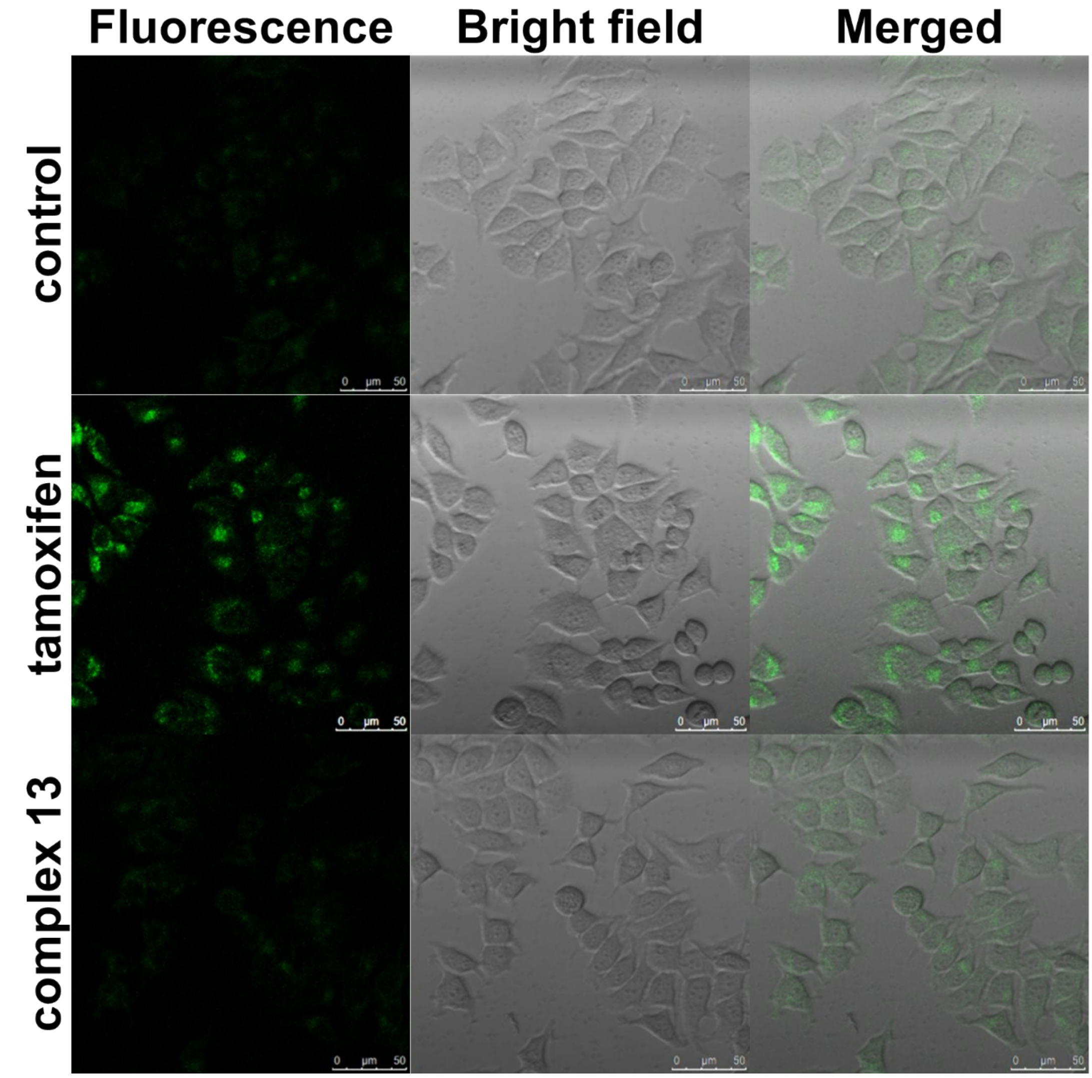

Figure S7. Confocal images of MDC stained MCF-7 cells upon treatment of 10 μM tamoxifen or 0.1 μM complex **13**.

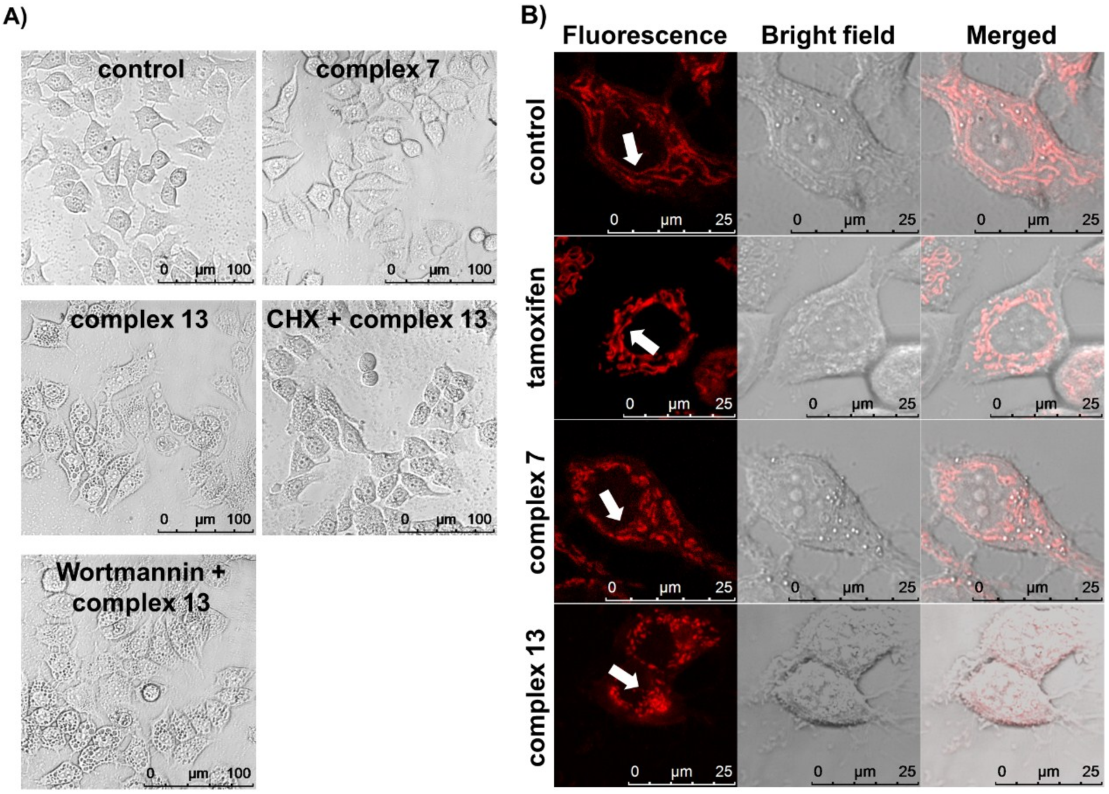

Figure S8. A) Confocal images of MCF-7 cells treated with 9 μM complex **7** or 3 μM complex **13** for 4 h with or without the pretreatment of inhibitors. B) Morphology of mitochondria in MCF-7 cells upon treatment of 10 μM tamoxifen, 0.5 μM complex **7**, or 0.1 μM complex **13**.

References

(1) W. L. Man, H. K. Kwong, W. W. Y. Lam, J. Xiang, T. W. Wong, W. H. Lam, W. T. Wong, S. M. Peng and T. C. Lau, *Inorg. Chem.,* 2008**, 47**, 5936-5944.

(2) W. L. Man, T. M. Tang, T. W. Wong, T. C. Lau, S. M.Peng and W. T. Wong, *J. Am. Chem. Soc.*, 2004, **126**, 478-479.

(3) A. Altomare, G. C., C. Giacovazzo, A. Guagliardi, M. Burla, G. Polidori, M. J. Camalli *Appl. Crystallogr.* **1994,** *27*.

(4) P. T. Beurskens, G. A., G. Beurskens, W. P. Bosman, R. de Gelder,. R. Israel, J. M. M. Smits *Technical Report of the. Crystallography Laboratory*; University of Nijmegen: 1999.

(5) Crystal Structure, Single Crystal Structure Analysis Software, version 3.5.1; Rigaku/MSC Corporation: The Woodlands, Texas, USA, Rigaku, Akishima, Tokyo, Japan, 2003; D. J. Watkin, C. K. Prout, J. R. Carruthers, P.W. Betteridge, Crystals, Chemical Crystallography. Lab: Oxford, UK, 1996; issue 10.
